# Health Education England, Local Education and Training Boards (LETBs) and reform of healthcare education: implications for surgical training

**DOI:** 10.1186/1471-2482-15-3

**Published:** 2015-01-15

**Authors:** Jonathan RL Wild, J Edward F Fitzgerald, Andrew J Beamish

**Affiliations:** Association of Surgeons in Training, 35 - 43 Lincoln’s Inn Fields, London, WC2A 3PE United Kingdom

**Keywords:** Education, Training, Surgery, Reform, NHS, Workforce

## Abstract

**Background:**

National Health Service (NHS) reforms have changed the structure of postgraduate healthcare education and training. With a Government mandate that promotes multi-professional education and training aligned with policy driven initiatives, this article highlights concerns over the impact that these changes may have on surgical training.

**Discussion:**

The creation of Health Education England (HEE) and its local education and training boards (LETBs), which are dominated by NHS healthcare providers, should result in greater accountability of employers in workforce planning, enhanced local responsibility and increased transparency of funding allocation. However, these changes may also create a potential poacher-turned-gamekeeper role of employers, who now have responsibility for junior doctors’ training. Analysis of LETB membership reveals a dearth of representation of surgeons, who comprise only 2% of board members, with the input of trainees also seemingly overlooked. A lack of engagement with the LETBs by the independent sector is a concern with increasing numbers of training opportunities potentially being lost as a result.

The new system also needs to recognise the specific training needs required by the craft specialties given the demands of technical skill acquisition, in particular regarding the provision of simulation training facilities and trainer recognition. However, training budget cuts may result in a disproportionate reduction of funding for surgical training. Surgical training posts will also be endangered, opportunities for out-of-programme experience and research may also decline and further costs are likely to be passed onto the trainee.

**Summary:**

Although there are several facets to the recent reforms of the healthcare education and training system that have potential to improve surgical training, concerns need to be addressed. Engagement from the independent sector and further clarification on how the LETBs will be aligned with commissioning services are also required. Surgical training is in danger of taking a back seat to Government mandated priorities. Representation of trainees and surgeons on LETB committees is essential to ensure a surgical viewpoint so that the training needs of the future consultant workforce meet the demands of a 21^st^ century health service.

## Background

There have been significant changes in the healthcare education and training architecture over the past eighteen months, brought about as part of the coalition Government’s wide-ranging National Health Service (NHS) reforms [1]. In this article we attempt to provide an overview of the changes, highlighting the additional challenge these reforms may have to the funding of postgraduate medical education and surgical training. With a Government mandate that promotes multi-professional education and training aligned with policy driven initiatives, there are concerns over the composition of newly formed local education and training boards (LETBs). Specifically, there is a lack of representation of secondary care clinicians, especially surgeons, with the input of trainees also seemingly overlooked. We have therefore also sought to examine the composition of the LETB governing boards in order to determine the individuals who have been appointed to control the local budget for education and training and which disciplines have their interests promoted at the LETB board level.

## Discussion

### Reasons for change

Although changes to education and training were initially branded an afterthought to the Government’s wider reforms [2] a policy framework for a new approach to education and training of the healthcare workforce was set out in *Liberating the NHS: Developing the Healthcare Workforce – From Design to Delivery* [3], and from April 2013 these plans came into effect. The Government claims that reform of the healthcare education and training system has been necessary in order to provide a workforce that is capable of delivering 21^st^ century care. With a stagnant health service budget the NHS is faced with an ageing population, increased demands for novel treatments, changing patterns of care, an expectant public and concerns over quality and patient safety following the reports into care failings by Robert Francis QC [4] and Sir Bruce Keogh [5]. Proposals in *Shape of Training* – a recent review of postgraduate medical training led by economist Professor Greenaway [6], also outline the need for training to be changed to ensure future medical graduates can provide general care in broader-based specialties. Therefore, in order to serve the needs of patients and the public with a workforce that has the knowledge and skill-set to provide safe, effective and compassionate care, the reforms set out the need for greater efficiency and transparency in the investments made in education and training with a system that has national oversight and is flexible for local workforce needs.

### Health Education England

**Figure 1:**
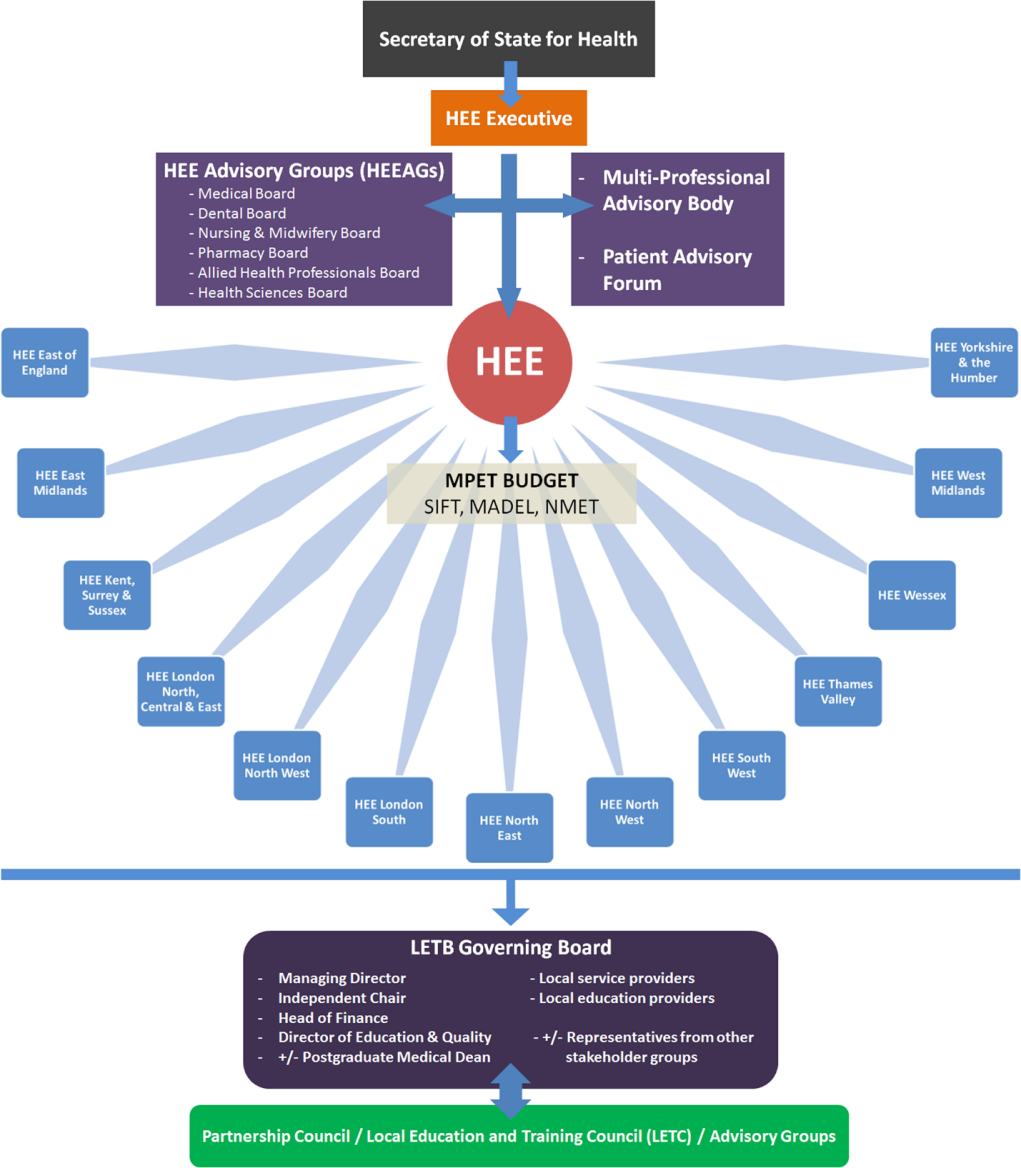
**Health Education England and its LETBs.**

The main elements of the healthcare education and training reforms have been the formation of Health Education England (HEE) and its 13 LETBs, which have absorbed the function of the postgraduate deaneries and have replaced education and training functions of the former 10 regional strategic heath authorities (see Figure 1).

HEE is a statutory body with the responsibility to provide national leadership and oversight on strategic planning and development of the healthcare workforce in response to workforce intelligence and changing patient needs [7]. It is the first time a single organisation has had the responsibility for the education, recruitment, training and development of the entire NHS healthcare workforce. As part of HEE’s advisory structure, there are six advisory groups (HEEAGs) who will promote the interests of the medical, dental, nursing, pharmacy, healthcare science and allied healthcare professional groups. The Medical Advisory Group provides national level representation of the medical profession and is responsible for medical recruitment, supporting curriculum-based training, ensuring there is capacity in the NHS to deliver training to a high standard and promotes academic disciplines.

HEE will develop an allocation policy to ensure efficiency and transparency of the flow of funding to the LETBs of the £4.8 billion multi professional education and training (MPET) budget to the LETBs, which comprises of the service increment for teaching (SIFT), the medical and dental education levy (MADEL) and the non-medical education and training (NMET) funding streams. Initially a tariff based system for education and training will be implemented, with the longer-term possibility for MPET to be funded by a training levy on all providers, which would allow the collection of a contribution from independent providers of healthcare who employ and benefit from staff trained by the NHS. Mechanisms have been developed to ensure efficiency and transparency of the flow of funding for education and training from HEE to its LETBs, and an Education and Outcomes Framework (EOF) is being developed, which will set clear outcomes for the education and training system and the consequential impact on the patient experience, care and safety [8] enabling the allocation of education and training resources to be linked to quantifiable quality outcomes [9]. HEE is therefore likely to have greater influence than Medical Education England (MEE), which it has replaced, as it acts as the body responsible for improving the quality of education and training in addition to controlling the purse-strings. However, the last minute cuts to the MPET budget by the Department of Health, where HEE discovered they were to receive £150 million less than expected for 2012/13, [10] mean that any new found influence may well be dampened.

### The Government’s mandate for education and training and an integrated multi-disciplinary training

The recently published Government mandate to HEE [11] has been dubbed the “blueprint for NHS staff training” [12]. It is aligned with national NHS priorities to tackle preventable deaths, support people living with chronic health problems, to increase patient access to psychological therapies and to improve the diagnosis and management and care for people with dementia, including providing dementia awareness for all frontline NHS staff. General Practitioners (GPs), midwives, pharmacists, healthcare assistants (HCAs), community nurses and health visitors are the priority target staff groups, with an emphasis on a shift to community care. In response to the *Francis Report*, training is to be focussed on promoting a culture of caring with those who embark on a nursing degree to serve a year as a HCA and there are moves to ensure clearer and more effective career paths for HCAs including routes into senior care assistant roles and professional training. There appears to be a firm focus on integrated multi-disciplinary training, with the mandate stating that “where appropriate [training] should incorporate working in multi-skilled teams reflecting care pathways rather than exclusively professional groupings” [11].

### Local education and training boards

The LETBs offer a forum for healthcare providers and professionals to improve the quality of education and training outcomes within their local area and ensure the supply of the local NHS healthcare workforce, whilst supporting national workforce priorities. The governing board of each LETB determines local arrangements for the commissioning of education and training through allocation of its share of the MPET budget. Some LETBs have sub-committees, such as Local Education Training Councils (LETCs) and professional advisory groups in order for the LETB to engage with stakeholders. However, with no standard model for LETBs to follow there is the potential for regional variability in how each LETB functions, which is perhaps why 9 out of the 13 LETBS have conditions imposed on their authorisation, including involvement of HEE to determine the make-up of their boards [13].

### Who sits on the LETB boards?

To determine who sits on the 13 LETB boards and which professional interests are represented we obtained lists of LETB board members via the HEE websites, as of June 2013. If details were not available online the LETB was contacted directly for the information on the role and background of LETB board members.

Each LETB has a Managing Director, a Director of Education and Quality (DEQ), who is responsible for the quality management of education and training programmes commissioned or provided by the LETB, both employed by HEE, an Independent Chair, whose role is to ensure conflicts of interest do not diminish the LETBs effectiveness and a Head of Finance [14]. Postgraduate Medical Deans are also LETB board members, with some now undertaking the role of DEQ (see Figure 1).

The majority (59%) of the 231 LETB governing board members are representatives of NHS health service providers (see Table 1). In the main these provider representatives are chief executives and senior managers of local NHS trusts, 30% of whom have a background in nursing. Education providers represent 12% of LETB members and just 10% primarily represent the training interests of healthcare professionals, including the postgraduate deans and DEQs. Only one LETB, HEE North Central and East London, has a trainee, medical student and patient representative on their board. Of the 66 medically qualified LETB board members, 30 (45%) represent NHS service providers, with the majority of these (73%) being GP providers (see Table 2). Broken down by specialty, there are only 4 surgeons, representing under 2% of total LETB governing board membership. Thirty four (52%) of those LETB board members who are medically qualified are general practitioners with the acute medical specialties represented by 17 (26%) of these medically qualified board members.

**Table 1:** **The proportion of LETB board membership revealing the LETBs are dominated by service providers and administrators**

LETB	LETB board membership						Total number
	Health service provider	Education provider	Administrator	Healthcare professional representative	Trainee/medical student	Patient	
**East Midlands**	10(71%)	1(7%)	2(14%)	1(7%)	0(0%)	0(0%)	14
**East of England**	11(65%)	2(12%)	3(18%)	1(6%)	0(0%)	0(0%)	17
**Kent Surrey Sussex (KSS)**	11(65%)	3(18%)	2(12%)	1(6%)	0(0%)	0(0%)	17
**London(NCE)**	7(41%)	2(12%)	3(18%)	2(12%)	2(12%)	1(6%)	17
**London(NW)**	7(47%)	2(13%)	3(20%)	3(20%)	0(0%)	0(0%)	15
**London(S)**	9(53%)	2(12%)	3(18%)	3(18%)	0(0%)	0(0%)	17
**North East**	14(61%)	4(17%)	4(17%)	1(4%)	0(0%)	0(0%)	23
**North West**	12(57%)	3(14%)	4(19%)	2(10%)	0(0%)	0(0%)	21
**South West**	9(56%)	1(6%)	3(19%)	3(19%)	0(0%)	0(0%)	16
**Thames Valley**	10(63%)	1(6%)	3(19%)	2(12%)	0(0%)	0(0%)	16
**Wessex**	13(65%)	2(10%)	3(15%)	2(10%)	0(0%)	0(0%)	20
**West Midlands**	10(59%)	2(12%)	4(23%)	1(6%)	0(0%)	0(0%)	17
**Yorkshire & Humber**	12(62%)	3(14%)	3(14%)	2(10%)	0(0%)	0(0%)	21
**Total**	136(59%)	28(12%)	40(17%)	24(10%)	2(0.9%)	1(0.4%)	231

Those primarily representing the training needs of healthcare professionals are in the minority.

**Table 2:** **Breakdown of the 66 medically qualified LETB board members by specialty reveals that the craft specialties are under-represented**

Specialty	Number of medical qualified representatives on LETBs	Subspecialty
General practice	34 (52%)	-
Medicine	17 (26%)	Nephrology ×4
		General medicine ×3
		Care for the elderly ×2
		Respiratory ×2
		Cardiology ×2
		Rheumatology ×2
		Gastroenterology ×1
		Paediatric medicine ×1
Surgery	4 (6%)	Urology ×2
		Breast surgery ×1
		Vascular surgery ×1
Other	11 (16%)	O + G ×3
		Pathology ×2
		Psychiatry ×2
		Anaesthetics ×1
		Microbiology ×1
		Clinical chemistry ×1
		Reproductive Medicine ×1

### Provider led commissioning of education and training - poachers-turned-gamekeepers?

It is clear that the LETBs reflect the Government’s ambition that they are very much service provider led. Education providers, the local universities and medical schools are also entitled to serve on the board [14] although this does not appear to be the case in all LETBs. It is the healthcare service providers who are therefore chiefly responsible for educating and training doctors, dentists, nurses and all other allied healthcare professionals at a local level in England and now take the lead in the planning and development of the local workforce.

With the LETBs being dominated by health service providers there is the danger of conflict of interest with a potential poacher-turned-gamekeeper role of employers, who now have responsibility for junior doctors’ training [2]. Trusts often provide their own training courses so there is a real potential they could now commission from themselves and also be involved in quality assurance of the training they provide. Similarly, there are also examples of individual LETB board members having additional roles in private health education providers, although the conflicts of interest associated with the LETBs do not appear to be as “rife” as on the new clinical commissioning groups (CCGs), where a third of GPs on the CCG boards have financial links to private providers from whom the CCG board may commission patient services [15].

It is also unclear how the service commissioning processes for CCGs will be aligned with the commissioning of education and training by the LETBs. Certainly, education and training should be mandatory for a commissioning contract to be awarded. However, it seems somewhat contradictory that, despite LETBs being primarily provider-led, CCGs are not permitted to have those who work in local providers sit on their boards [16]. The reasoning behind the exclusion of those who work in local providers, such as consultants, from CCGs is potential conflict of interest, which, rather rigidly, takes priority over local knowledge and expertise [17]. It is also not clear how training would be protected in the event that a hospital-based training provider with an existing contract with a LETB has its service de-commissioned by a CCG. Further clarification is required on how CCGs and LETBs will work together.

### A lack of engagement by the independent sector

It is disappointing to reveal that no independent sector providers are yet represented on the LETBs, despite it being a duty of all providers of public funded health services to be members of the LETB governing board [18]. The independent sector is playing an increasing role in the provision of elective surgery, with one fifth of NHS elective hip and knee surgery currently performed in the independent sector [19]. As numerous aspects of surgical patient care shift towards the independent sector centres, in particular the day case and low risk procedures that are often more suitable training cases, independent sector engagement with the LETBs is important to prevent training opportunities being lost.

### Clinician and trainee involvement with LETBs

It is essential to involve clinicians in the decision-making involving education and training and workforce planning. Clinicians are best placed to know what courses and training are required for specific postgraduate programmes as well as advising on appropriate quality assurance. Taking into account the views of doctors and other professionals in local plans is important for getting the content of such education and training right. Involvement will improve clinician confidence in the new arrangements [20]. Although amendments to the Care Bill now require that the boards of LETBs include persons with clinical expertise, the majority of board members with clinical backgrounds are in management roles, primarily representing providers, rather than the training and education needs of doctors. With only four surgeons currently involved at LETB board level, it is also a concern that in many LETBs the surgical voice may be lost amongst other competing disciplines. It is therefore important that surgeons, if not represented at board level, are willing engage with the LETB advisory groups in order to promote the needs of surgical training locally.

Misgivings over a lack of representation of trainees and students on LETBs have been recently raised by the British Medical Association (BMA) [21]. It is imperative that, as individuals for whom the training system is provided, opportunities should be provided for trainees and students to be involved in shaping the local training and education system, if not at board level, then certainly in LETB advisory or user groups. A similar argument can be extended for more involvement of patient representatives in the LETBs.

Going forward, the regionalised decision-making responsibilities of LETBs may allow greater flexibility and response to local training needs. However, this comes at a cost, with a real risk of regional inequalities and widening variation in training provision. Countering this will be more difficult, with individual trainee or patient representation (where present) potentially weakened by those decentralised decision making responsibilities.

### Implications for surgical training and education

Trainee productivity contributes significantly to the healthcare service income and has been estimated to earn NHS Trusts far more than trainees’ salaries cost, with relatively little education and training commitment in return from the employer [22]. Despite this, there is concern that cuts to the MPET budget and a drive for multi-professional education and training will result in a disproportionate reduction of funding for medical education and training. Training posts will therefore be endangered and opportunities for out-of-programme experience and research may decline.

With surgical training being of a greater duration than many specialties, surgical trainees will be affected more than other specialties by further reductions in funding for education and training. Over the last decade, The Association of Surgeons in Training (ASiT) has investigated the cost of surgical training to trainees themselves, highlighting a sustained shift of training costs away from the health service onto trainees [23–25]. On a background of rising graduate debt a decline in educational funding is set against above inflation rises in the cost of surgical training, examination and regulation. This includes multiple professional fees, such as the contentious Joint Committee of Surgical Training (JCST) fee that surgical trainees are required to pay in order to support the administration of their own training. Study budgets continue to be top-sliced to support local curriculum delivery and are clearly insufficient to support trainees undertaking mandatory courses and exams required for progression.

The current surgical training system therefore requires increasing personal investment in order to succeed and many trainees, unless independently wealthy, are incurring significant debt which is detrimental to the experience of surgical training. This situation also flies in the face of initiatives that aim to widen the access to surgical careers for individuals from less privileged backgrounds and may limit the diversity of entrants into surgical training. It is a concern that in the UK surgery is becoming a less popular career choice and we may also see a “brain-drain” in coming years as surgical trainees seek better training and financial prospects abroad [24]. Given the year-on-year increase in requests for ‘Certificates of Good Standing’ from the General Medical Council (GMC), enabling doctors to practice overseas, there is limited evidence this may already be occurring [26].

HEE’s strategy appears to be focussed on community care and long-term conditions (mainly non-surgical disease processes) and as a result the future needs of surgical training may take a back seat to HEE mandated priorities that are influenced by wider policy issues. One such example is the need to invest in simulation training which is increasingly being utilised in surgical training. Simulation training is now seen as a requirement of surgical training programmes in order to compensate for reduced working hours and enhance patient safety. However, high-quality simulation training is expensive and requires consultant trainers with time dedicated for simulation training. In addition to significant investment in local simulation facilities, the successful integration of simulation into local surgical training programmes requires surgical trainers to be recognised appropriately in consultant job plans [27]. It is reassuring that the JCST have been invited to participate in a HEE working group on technology-enhanced learning (TEL) [28] and it is imperative that appropriate investment into simulation facilities and trainers is provided.

The new system, both national and regional, needs to recognise the specific educational and training needs required by the craft specialties given the demands of technical skill acquisition [29]. There is a lack of evidence supporting the emphasis on a much greater multi-professional approach to training and education. While there may be opportunities for inter-professional learning in areas such as non-technical skills, leadership and management, a uni-professional approach is required as different professions have different requirements. Surgical trainees need time to understand the unique demands and requirements of their own profession in order for them to contribute effectively in an integrated team and to appreciate the skills and expertise of other team members. Multi-professional learning is better suited to continued professional development once trained.

## Summary

There are several facets to the recent reforms of the healthcare education and training system that have potential to improve surgical training. Greater accountability of employers in workforce planning and development, enhanced local responsibility and increased transparency of the MPET funding allocations are welcomed in order to provide a system that is built around the present and future needs of the patient. Plans to incentivise training via the introduction of a tariff-based system and a training levy on all healthcare providers are long overdue and are especially welcomed. However, there are several concerns, in particular over conflicts of interest with the with the local healthcare service providers who form the majority of the LETBs. Involvement from the independent sector and further clarification on how the LETBs will be aligned with CCGs are also required. Given that surgical training is in danger of taking a back seat to HEE mandated priorities, representation of trainees and surgeons on the LETB committees and boards is essential to ensure a surgical voice is heard and that the training needs of the future consultant workforce are met. It is therefore imperative that there is on-going engagement and collaboration between HEE and the surgical Royal Colleges, JCST and other surgical professional associations, including trainees, to ensure that best use is made of surgical innovations and that there is sufficient investment in simulation facilities and appropriate surgical trainer recognition to ensure that the surgical workforce is prepared for the demands of a 21^st^ century health service.

## Authors’ information

JRLW is a speciality registrar in general surgery in Yorkshire and the Humber and Council member for the Association of Surgeons in Training.

JEFF is a speciality registrar in general surgery in London and Past President of the Association of Surgeons in Training.

AJB is a speciality registrar in general surgery in Wales and Immediate Past President of the Association of Surgeon in Training.

## Abbreviations

ASiT: Association of surgeons in training
BMA: British medical association
CCGs: Clinical commissioning groups
DEQ: Director of education and quality
EOF: Education and outcomes framework
GMC: General medical council
GPs: General practitioners
HCAs: Healthcare assistants
HEE: Health education England
HEEAGs: Health education England advisory groups
JCST: Joint committee on surgical training
LETBs: Local education and training boards
LETCs: Local education and training councils
MADEL: Medical and dental education levy
MPET: Multi professional education and training
NHS: National health service
NMET: Non-medical education and training
SIFT: Service Increment for teaching
TEL: Technology-enhanced learning.
